# Conflict, forced displacement and health in Sri Lanka: a review of the research landscape

**DOI:** 10.1186/1752-1505-8-22

**Published:** 2014-11-03

**Authors:** Chesmal Siriwardhana, Kolitha Wickramage

**Affiliations:** Department of Psychological Medicine, Institute of Psychiatry, King’s College London, London, UK; Faculty of Medical Sciences, Anglia Ruskin University, Chelmsford, UK; Institute for Research & Development, Sri Jayawardenepura Kotte, Sri Lanka; School of Public Health and Community Medicine, Faculty of Medicine, University of NSW Sydney, Sydney, Australia; International Organization for Migration, Colombo, Sri Lanka

**Keywords:** Sri Lanka, Civil conflict, Health, Health research, Health systems, Post-conflict, Mental health, Internal displacement

## Abstract

Sri Lanka has recently emerged from nearly three decades of protracted conflict, which came to an end five years ago in 2009. A number of researchers have explored the devastating effect the conflict has had on public health, and its impact on Sri Lanka’s health system - hailed as a success story in the South Asian region. Remarkably, no attempt has been made to synthesize the findings of such studies in order to build an evidence-informed research platform. This review aims to map the ‘research landscape’ on the impact of conflict on health in Sri Lanka. Findings highlight health status in select groups within affected communities and unmet needs of health systems in post-conflict regions. We contend that Sri Lanka’s post-conflict research landscape requires exploration of individual, community and health system resilience, to provide better evidence for health programs and interventions after 26 years of conflict.

## Introduction

### Country facts

Sri Lanka is categorized as a lower middle-income country (LMIC) [1] with a population of 21 million [2]. The country has a multi-ethnic society where the majority (74%) are Sinhalese, 18% are Tamil and 7% Moor. The remaining 1% consists of Burgher, Malay and Veddas. Current literacy rates are 92.5% in males and 87.9% in females with an average life expectancy of 76.3 years [2].

### Health systems and services in Sri Lanka

The public health achievements of Sri Lanka have been hailed as a success story by the World Health Organization (WHO) and it currently has the best health indicators in the South Asian region [3]. The crude death rate is 6.0 per 1000 population and maternal mortality is 22.3 per 100,000 live births [4]. Over 90% of current live births occur in a government hospital settings in the country, and the immunization coverage is around 99% [5]. Sri Lanka has a free-of-charge health care system, heavily subsidised by the government. However, private health care is also readily available. The country's health expenditure is 4.1% of the total government expenditure with per capita health expenditure around 400 USD [4]. Sri Lanka has 0.680 general physicians per 1,000 residents compared to 2.790 physician density of the United Kingdom [6]. Mental health has been recognized as a key component of primary health care in Sri Lanka but mental health services are mostly based around institutional care, lacking a public health approach [3]. According to current figures, the country has a total of 2031 psychiatric hospital beds and only around 48 specialist psychiatrists for over a 20 million population [7].

## Conflict and displacement in Sri Lanka

Since achieving independence from Britain in 1948, Sri Lanka experienced two major armed conflicts that effectively impacted on the whole country. The Janatha Vimukthi Peramuna (JVP), a leftist organization involving mainly Sinhalese youth, led two insurgencies in the southern part of the country, first in 1971 and later between 1987-1990 [8]. Around 60,000 people were killed during these episodes, and included JVP cadres, members of the armed forces and civilians [8]. While major population movements were not triggered by these events, a considerable number of individuals and families were displaced from their homes, mainly due to prosecution by various factions involved.

The other major armed conflict to impact the Sri Lankan population was the protracted war between the Sri Lankan government and the Liberation Tigers of Tamil Eelam (LTTE), a militant organization aiming to create an autonomous Tamil state in the Eastern and Northern provinces of the country. The armed conflict which started in 1983 came to an end in May 2009, with the military defeat of the LTTE by Sri Lankan armed forces [9, 10]. During the 26 years of prolonged conflict, more than 100,000 people of all ethnicities are estimated to have died, while hundreds of thousands have been injured [9, 10]. The three-decades of conflict was also the key driver of internal and external displacement in Sri Lanka, displacing approximately 800,000 people at its peak in 2001, mainly from the Northern and Eastern provinces [11]. Current estimates indicate that approximately 90,000 (0.4% of the total population) people are internally displaced in Sri Lanka [11]. There are also an estimated 73,000 Sri Lankan refugees living in 112 camps in the southern Indian state of Tamil Nadu with a further 34,000 outside the camps [12]. However, these numbers can vary as refugee population tend to grow in size and has a high level of undocumented migration. Throughout the period of conflict, the numbers of Sri Lankan asylum seekers to countries such as UK, Canada and Australia has been increasing, with their irregular migration contributing especially to mental health problems [13, 14].

Internal displacement in Sri Lanka has shown a highly fluid character, ebbing and flowing with the nature of the conflict and its severity [9]. Populations experiencing conflict-driven internal displacement had to undergo highly traumatic migration episodes, and some populations has had endure displacement periods lasting several decades. These experiences stand to strongly affect the long-term health of displaced populations, and especially present a high risk of developing mental disorders.

Whilst recognizing the multiple research efforts that have been undertaken through three decades of conflict, there has been little attempt to synthesize research findings on the health impact of conflict affected populations. We aim to investigate the ‘research landscape’ and take stock of the emergent findings that supports establishing an evidence-informed research platform. This paper therefore will explore the health impact of conflict among Sri Lankan population through an examination of the currently available research evidence, with an emphasis on conflict-driven internal displacement and mental health.

## Review methodology

For this purpose, a literature search was carried out using Pub Med, BIOMED CENTRAL, Cochrane Library, OVID, Psych Info, Google Scholar, Embase, Sri Lanka Journals Online and other main data sources for English language articles originating from Sri Lanka, using the key words; Sri Lanka; conflict; health; physical health; mental health; internal displacement. These key words were used in different combinations to maximise the results. Studies using quantitative, qualitative or mixed-methods were included. Although the focus was to find empirical evidence, relevant reviews were considered.

No limits were imposed on publication dates. Studies on refugee populations with a Sri Lankan origin were excluded to keep the review focus on affected populations within the country. Articles without a direct link to conflict or conflict-affected populations were also excluded. Although this is not a systematic review, the search strategy followed similar established procedures in selecting relevant studies. A narrative synthesis was then conducted.

## Physical health impact

Conflict-driven forced displacement precipitates physical ill health among affected populations [15], during pre-flight, flight and post-flight periods [16]. In addition, conflict situations increase public health problems, compounded by existing health disparities [15, 16]. In the Sri Lankan conflict setting, published studies provide evidence of health system disruption, public health issues affecting displaced populations, increased mortality, morbidity and disease burden (quantified by quality adjusted life years - QALYs or disability adjusted life years - DALYs), disruptions of service provision in affected regions and problems with post-conflict health needs [17, 18].

In the Northern province, higher than national average maternal and neonatal mortality rates were reported, with high levels of low birth weight babies being born and increased number of stillbirths [17–19]. A study conducted during the conflict (2004-2005) on reproductive health of women in six conflict areas of Sri Lanka showed higher levels of marriage at an early age, pregnancy at an early age, increased home births, low contraception and increased maternal mortality rates [20]. This study also highlighted that women in conflict areas faced a higher risk of sexual and physical abuse, and faced various barriers in receiving adequate health care and services [20]. Furthermore, antenatal care utilization was shown to be significantly poor for women whose families were affected by conflict in Northern Sri Lanka or living in an active conflict zone in a study conducted in 2009 [21].

Another critical area of public health importance in conflict situations is the risk of infectious disease spread. Several studies done in conflict areas and among displaced populations in Sri Lanka showed a marked increase of infectious disease [17]. Acute disease outbreaks are often common within displaced camp settings due to inadequate water and sanitation facilities coupled with large populations living in close proximity within temporary accommodation/tent facilities. A large scale Hepatitis A outbreak was reported in internally displaced camps (IDP) camp settings in Vavuniya district in the aftermath of the 2009 conflict [22].

Protracted conflict also results in disrupted or fractured disease control programs. While malaria has been effectively controlled in most areas of Sri Lanka (incidence declined by 99% and only 124 indigenous cases reported in 2011), malaria incidence was reported to be much higher in the conflict regions [23]. However, even during the conflict and especially in the post-conflict period, the incidence rate has been rapidly decreasing, due to the strong epidemiological surveillance, preventive and treatment systems activated in these regions [24]. Sri Lankan asylum-seekers from Northern Province continue to pose a new and emerging threat of malaria re-introduction as they return from failed asylum bids, usually as a result of irregular migration through endemic areas in West Africa [25]. Sri Lanka’s National Programme for Tuberculosis Control and Chest Diseases (NPTCCD) also reports the prevalence of TB to be high among returning Sri Lankan refugees who were living in refugee camps in India. TB detection rate among such groups being resettled in mostly Northern districts is at 936 per 100,000 population, a higher rate than the national figure of 46 cases per 100,000 [26]. Since the end of conflict, there has been a sustained effort made by the NPTCCD in scaling up TB control activities in resettlement areas of the Northern and Eastern provinces [26]. Other infectious diseases such as leishmaniasis have also affected populations in such regions [27].

In conflict-driven displacement situations, there is an increased risk of physical trauma to civilian populations. In the Sri Lankan context, physical trauma resulted due to direct attacks from warring parties, getting caught in artillery or shelling and being injured by bombs, land mines, improvised explosive devices (IED), anti-personal mines and other forms of exploding munitions [28, 29]. Physical injuries included shrapnel wounds, limb amputations, visceral wounds and other forms of damage [28–30]. Armstrong et al. [31] describe high volumes of orthopaedic trauma seen among the IDP population at the end of the Sri Lankan conflict in 2009. A team affiliated with Medecins Sans Frontieres (MSF) conducted a rehabilitation programme for IDPs with spinal cord injury in the Northern province, working together with staff from the Ministry of Health, Sri Lanka. They provided nursing, physiotherapy, mental health care and vocation rehabilitation through the programme, and highlight the lack of adequacy in services for IDPs disabled with such high-intensity physical trauma [31]. The high rates of physical trauma in these regions has seen a dramatic drop, mainly due to the clearance of landmines after the end of conflict [32]. However, personnel involved in landmine clearing operations have been reportedly injured due to accidents when handling explosives [32].

In addition, high levels of malnutrition and disability has been reported among displaced populations [33–35]. However, accurate figures on the levels of malnutrition or disability are not available on populations displaced at the end of conflict. Nagai et al., [18] explored the 20-year trends in health service provision and health status in the conflict-affected Northern province and concluded that those outside the conflict region had a very little understanding about the actual health needs in affected areas. They further stated that strategic development and allocation of human resources for health is required to rebuild the health service systems in the Northern province. Empowering local communities and a systematic mental health strategy is also advocated [18]. Although more access has been gained by the Sri Lankan Ministry of Health into these areas since the end of conflict, a concerted effort at conducting an accurate census is yet to materialise. A study exploring immunization status among IDP children in post-conflict areas during resettlement found that while the infant vaccination coverage in the war-torn Kilinochchi district was not different to other Sri Lankan districts, there was a marked disparity in age-appropriateness of the vaccination coverage in Kilinochchi [36]. Although the coverage levels were similar to other post-conflict settings, authors point out that Sri Lanka needs to focus on ensuring the continuation of full vaccination in the post-conflict region, which may gets disrupted due to resource availability and service priority changes that can happen in the post-conflict period [36].

The above evidence shows various facets of the impact created by the Sri Lankan conflict on the physical health of affected populations, especially IDP. The evidence highlight the lack of up-to-date epidemiological data, inadequacies in service provision and lack of access to care for affected populations. However, during the last five years since conflict cessation, a large amount of programmes including immunization surveys, psychosocial assistance and other health system rebuilding work has been carried out by the Sri Lankan Ministry of Health and other agencies [37, 4]. This work is especially relevant in the current post-conflict era, when a comprehensive restructuring of the health system is required to address burgeoning health needs of the returning IDP populations [33].

## Mental health impact

The Sri Lankan civil conflict has had an enormous impact on the mental health of the country's population, especially in the areas of Northern and Eastern provinces. This impact has been compounded by the 2004 tsunami striking many conflict areas and causing death and destruction among communities already burdened by many years of war-related trauma [9, 38]. Children and adults from every ethnicity, religion and socio-economic background have faced conflict-related mental health issues in the country as individuals, families and communities.

### Collective trauma

Somasundaram [9] terms the disruption of family and community structure and destruction of the social fabric, networks, cohesion, and social capital as 'collective trauma. Somasundaram and colleagues have conducted several studies mainly among Tamil populations living in and/or displaced from affected areas in the Northern province during the conflict and describe how the impact of war is hugely detrimental to the collective mental health of affected communities [39–41]. These studies are mostly qualitative in nature and used a mix of anthropological, ethnographic and health research methodology.

A study conducted in 2007 among Tamil communities in the Northern province concludes that conflict had created fundamental changes in family and community dynamics, leading to increased psychosocial problems in a collective sense [39]. Another more recent study explored psychosocial status among displaced populations in the Vanni area of Northern Sri Lanka. These populations were displaced at the end of the conflict in 2009, and had endured extreme hardship during the closing stages of the war. The findings show collective symptoms of decreased psychosocial health and the author recommends interventions that target memory healing and psychosocial regeneration of families and communities in the post-conflict rebuilding and rehabilitation phase [40]. Another study conducted by Somasundaram & Sivayokan in 2012 among conflict-affected, former displaced communities in the Northern province showed that war-related complex psychosocial issues are preventing recovery [41]. They identified a range of issues linked to poor psychosocial health including unresolved grief, self-harm, suicidal ideations, insecurity, poverty, teenage pregnancies, gender based and domestic violence, neglect of the elderly and many others [41].

### Combatant mental health

Another area that has received some research attention, albeit limited, is the mental health of combatants involved in the Sri Lankan conflict. A study conducted in 2004 explored psychological status prevalence and the link with physical disability among a group of permanently disabled government soldiers, indicating that almost half of the sample had psychological distress (as measured by the General Health Questionnaire - GHQ) and one-third presented psycho-somatic symptoms [42]. Hanwella & De Silva conducted a study among Special Forces and regular regular force members of the Sri Lanka Navy in the close aftermath of the conflict (3 months after end of combat in May 2009) aiming to compare mental health problems between the two groups [43]. They found that while overall exposure to potentially traumatic experiences was high in both groups, and while Special Forces experienced a higher number of traumatic events, this group nevertheless had significantly less common mental disorder prevalence and less PTSD, compared to regular naval forces, and was attributed to the more close-knit, cohesive unit structures in Special Forces [43].

de Silva, Jayasekera and Hanwella [44] found a prevalence of 10.4% for multiple physical symptoms among the same cohort, and observed higher rates of symptom reporting among those with PTSD and common mental disorders. Another study has shown that heavy alcohol use was associated with poorer psychological health and functional impairment among military personnel in Sri Lanka [45]. A study looking at current smoking among military personnel showed a lesser prevalence than that of the general population and that smoking was significantly associated with combat experiences [46]. Two studies exploring mental health of soldiers with lower limb amputations and spinal injuries sustained during combat showed higher level of PTSD (41.7%) and other poorer mental health outcomes related to the injuries [47, 48].

Epidemiological or other evidence on mental health of rebel forces (LTTE members) are not available. However, child combatants and suicide bombers used by the LTTE in the civil conflict are thought to suffer from numerous combat related psychological issues such as somatisation, PTSD, depression, anxiety, behavioural and conduct disorders [49, 50]. Apart from combatants, humanitarian agency workers involved in providing relief services to conflict areas also experience conflict-related trauma. Lopes Cardoso et al. [51] explored factors affecting mental health of Sri Lankan nationals working for humanitarian organizations during the conflict and the post-conflict period, concluding that 19.0% of the 398 staff from 9 organizations reported symptoms of PTSD, 58.0% reported depressive symptoms and 53.0% with anxiety symptoms. Another study indicated higher levels of psychological distress among nurses caring for war victims in Sri Lanka [52]. While the level of stress was higher compared to civilian populations, the study found female nurses at a higher risk and being married or having children to be protective.

### Child and adolescent mental health

Research into the impact of Sri Lanka’s protracted conflict on child and adolescent mental health has been somewhat sparse in [53, 54]. A study on the effect of war related violence and violence inflicted on children and their families by Catani et al. showed that a relationship between war violence, family violence and PTSD in children [55]. In another study, more than half (58.0%) of the children reported exposure to armed conflict in various forms including experiencing direct violence, loss of family and displacement [46]. Fernando et al. [56] conducted a study to measure the impact of conflict (and tsunami) related stressors on the psychological and psychosocial functioning of adolescents in eastern Sri Lanka. The study examined relations between direct exposure to conflict stressors as well as to daily stressors which may be unrelated to emergency settings. The study concluded that daily stressors partially mediate the relation between exposure to conflict (and tsunami) and psychological outcomes among the studied group of adolescents. Abuse and material deprivation were relatively stronger predictors of PTSD than conflict (or tsunami) [56].

A national sample of children aged between 12-17 showed associations with exposure to conflict with poorer psychological outcomes and school absenteeism [53]. Another survey looking into trauma-related impairment in children from Northern and Eastern provinces of Sri Lanka showed a relationship between the total load of trauma experienced and performance or functioning [57]. As mentioned before, child combatants have shown increased war-related stress and other symptoms of psychological trauma [49]. However, specific epidemiological evidence relating to mental health of conflict displaced children and adolescents remains an unexplored research area.

### Forced internal displacement

Epidemiological studies exploring prevalence of mental disorders linked to conflict in Sri Lanka have been limited. Some studies have assessed trauma-related psychosocial status, anxiety or depression, albeit using small samples, and have focussed on developing culturally specific measures for Sri Lankan use [58, 38]. In addition, epidemiological evidence on mental health of conflict-driven IDP in Sri Lanka is sparse.

Somasundaram & Sivayokan reported an epidemiological survey on war trauma and its consequences on civilians, which was conducted in 1994 in a suburban area of Jaffna district in Northern Sri Lanka [59]. They reported high levels of war-related stress, PTSD (27.0%), somatization (41.0%) and major depression (25.0%). However, this study is limited by its sample size (101 in total) and findings cannot be generalized to other populations due to small cohort, sampling method and for being a highly localised study [59].

More recently, a larger and more representative cross-sectional household survey was carried out in Jaffna district of Sri Lanka, which aimed to understand the associations between mental health and displacement [10]. It was carried out between July and September 2009, few months after the cessation of conflict in the region. This study included around 1517 households in Jaffna along with 2 IDP camps. Out of a total of 1448 participants, 2.0% were current IDP, 29.5% were recently resettled IDP and the rest were non-IDP residents in the area. The study reported an overall prevalence of 7.0% PTSD, 32.6% of anxiety and of 22.2% depression among the population. In addition, findings showed that current IDP were more likely to have symptoms of PTSD, anxiety or depression compare to non-IDP residents. While the prevalence of mental disorder was associated with displacement status, it was also dependent on trauma exposure in this population [10].

Data from a cross-sectional patient morbidity register from primary care facilities in 4 district in the Northern province showed a major depression prevalence of 4.5% and a prevalence of 13.3% for mild depression among adult post-conflict population [60]. In comparison, the national prevalence of major and mild depression is 2.4% and 6.7% respectively [61]. The patient population numbering around 12,000 in this study included both resettled IDP and non-displaced groups and it was conducted between March-May 2013, approximately 4 years after the end of conflict. Findings also showed that females and older age groups have stronger associations with depression along with physical or cognitive impairments [60].

Another study explored common mental disorder (CMD) prevalence among a population of ethnic Muslims displaced over 20 years ago from the Northern province of Sri Lanka, who have been living in prolonged displacement in camps and resettlements in the North-western province [62]. It provides the only current evidence about the impact of prolonged displacement on mental health of conflict-affected populations in the country. The findings show that the CMD prevalence is higher (18.8%) than the national average (11.7%) [61]. The study also reports significant associations between unemployment, widowhood, food insecurity and mental health among the IDP [62]. However, it must be noted that associations reported in these studies must be treated with caution, as they may not be solely conflict-related or IDP-status related. Other region- or culture-specific factors may have played a role in higher levels of depression and other mental disorders in these populations, aggravated by the protracted conflict.

## Conclusions & future research directions

The small body of published literature about the impact of Sri Lankan civil conflict on various aspects of health show a clear paucity of epidemiological evidence. Studies on physical health, child and adolescent health and mental health are characteristically cross-sectional by nature, and are limited by their design, small sample sizes or highly localised populations of interest. In addition, very few studies have explored internally displaced populations. Only one study explored populations affected by prolonged internal displacement during the three decade Sri Lankan conflict [62]. Key focus areas of the studies included in the review are summarised in Table 1.

**Table 1:** **Key focus areas of the studies included in the review**

Key focus area		Studies
**Physical health**		
	***Maternal and child health***	Reilley et al. [17]; Simetka et al. [19]; Kottegoda et al. [20]; Sivaganesh & Senarath [21];
	***Infectious disease***	Reilley et al. [17]; Abeyasinghe et al. [24]; Dahanayaka et al. [22]; Wickramage et al. [25]; Semage et al. [27]
	***Physical trauma***	Goonetilleke [29]; Collie [28]; Covey [30], Armstrong et al. [31]
	***Nutrition and vaccination***	Rajapaksa et al, [35]; Jayatissa et al. [34]; Parameswaran & Wijesinghe [36]
**Mental Health**		
	***Collective trauma***	Somasundaram [39]; Somasundaram [40]; Somasundarama & Sivayokan, [41]
	***Combatants***	Somasundaram [49]; Kasturiarachchi & Jayawardana [42]; Gunawardena et al. [47]; Somasundaram [50]; Hanwella & de Silva [43]; de Silva et al. [46]; Hanwella et al. [43]; Abeysinghe et al. [24]; de Silva et al. [44]
	***Children and adolescents***	Chase et al. [54]; Catani et al. [55]; Elbert et al. [57]; Fernando et al. [56]; Siriwardhana et al. [62]
	***IDPs***	Husain et al. [10]; Siriwardhana et al. [53], Senarath et al. [60]
	***Other populations***	Somasundaram & Sivayokan [59]; IRD [61]; Fernando et al. [38]; Jayawickreme et al. [58]; Lopes Cardozo et al. [51]; Jayawardene et al. [52]; Knipe et al. [63]
**Health systems/policy**		Tribe [71]; Nagai et al. [18]; Siriwardhana et al. [53]

While the available evidence shows a high prevalence of mental health problems among conflict-affected populations in the North and East of Sri Lanka compared to other non-conflict areas [60–62], gaps of evidence are observed, especially regarding long-term mental health effects of forced internal displacement. Gaps also exist in understanding how health systems in conflict areas are recovering, and about the health of non-civilian populations, such as ex-combatants (both LTTE and the armed forces). Little is known or researched about their health status, modes of recovery, rehabilitation and/or re-integration to civilian life. However, prevalence of CMD such as depression and PTSD observed in conflict-affected populations in Sri Lanka is generally lower than that found in populations from other countries in Asia and Africa [62]. It must be noted that designs and measurements along with a host of cultural and social factors may account for the differences in prevalence of depression, PTSD or other CMD between Sri Lankan and other global populations. Studies included in our review also show variations in their design and mental health measurements, making accurate comparisons difficult in the absence of an existing baseline (except limited comparisons with the national mental health survey) [61].

Sri Lanka has been known for high suicide rates, and a recent historical cohort analysis highlighted the trends in suicide rates in the country [63]. However, data from conflict-affected regions were not captured and therefore not available due to non-systematic record keeping within disrupted health information systems. The population fraction (1.5% of the total country populations) of the conflict-affected areas were not considered to be sufficient to affect the overall analysis of suicide trends and the authors concluded that the civil conflict may have had a minimal impact on suicide trends [63]. However, further research is required to explore suicide trends in the post-conflict regions and associations with rapidly changing political, economic and social factors.

Protracted civil conflict erodes the fabric of social cohesion, dividing societies at the fault lines of prejudice [64]. The generational impacts war has on conflict affected societies, especially on children, can also extend well into periods of lasting peace [65]. The “fetal programming hypothesis” for instance suggests that conditions very early in human development, even *in utero*, can leave lasting imprints on a person's physiology and mental health - these imprints may ‘affect susceptibility to diseases’ with onsets potentially occurring many decades later [66–68]. An important factor neglected in assessing public health impact of the Sri Lankan conflict is the critical role of resilience in the recovery of individuals, communities, and disrupted health systems. Protracted conflicts systematically erode such inherent community capacities and challenges meaningful recovery. The small but growing body of research evidence on mental health and psychosocial interventions that promote individual and community resilience appear to hold considerable promise for promoting connectedness in the aftermath of conflict and disasters. Assessing interventional landscapes in Sri Lanka thus forms an important research priority [38, 69].

Post-disaster mental health interventions that extend beyond the provision of treatments for psychiatric morbidities and seek to strengthen social support have especially proven to be appropriate for many ethnic minority groups [32, 70]. Tribe [71], suggests that Sri Lanka would benefit from adapting a model of health pluralism as a more culturally appropriate alternative to western models of therapy for conflict and disaster related psychosocial issues in the population. Despite the long history of violent conflicts, the UN member states recognized the need for strengthening national health emergency and disaster management capacities and ensuring ‘resilience of health systems’ only at the 128^th^ Session of the World Health Assembly in January 2011. By ensuring the resilience of the health systems (critical for minimizing health hazards and vulnerabilities), delivering effective response and managing recovery in emergencies stand to become successful.

Number of studies cited in this review suggest the need to strengthen various aspects of the existing health system in the country, especially regarding the post-conflict regions and populations [10, 18, 20, 61]. The clear deficiencies and unmet needs in the health systems of post-conflict regions such as increased mortality and morbidity, lack of health workers, low access to services, low levels of knowledge in health issues, low levels of health promotion and awareness programmes require a sustained effort from responsible stakeholders including the government, regional governments, international organizations and academics [18, 72]. It must be noted however, that such deficits are in common to many post-conflict health systems across countries in Africa, Asia and Eastern Europe [73–75, 15]. A recent study examining Sri Lanka’s domestic legal framework in protecting the right to health of conflict displaced communities concluded that domestic laws provide sufficient provisions to enable health protection for IDPs [76]. Such provisions in the legal system can be utilised by the government to enforce a sustained drive to address gaps in health care provision to conflict-affected populations, and strengthen the health systems in post-conflict regions.

Health services and health systems research can be used in the Sri Lankan context to identify areas that require immediate or long-term solutions, such as human resources, disease burden and improving primary care. However, capacity building initiatives must be embedded within such research programmes to enable the development of the health workforce in post-conflict regions of Sri Lanka. It is critically important to establish collaborations between universities and academics from post-conflict areas and those from other parts of the country, as well as with international research community. Funding for research programmes that focus on post-conflict themes must be increased.

The research gaps highlighted in this review presents some key areas in Sri Lanka’s post-conflict research landscape that requires exploration of individual, community and health system resilience and recovery. Research identifying those factors and features of resiliency in Sri Lanka’s health system that enabled continued service coverage and function within resource limited conflict-affected areas during the protracted war should be conducted and may also be useful for other post-conflict countries.
